# Varsity Medical Ethics Debate 2015: should nootropic drugs be available under prescription on the NHS?

**DOI:** 10.1186/s13010-016-0041-5

**Published:** 2016-09-13

**Authors:** Emma Thorley, Isaac Kang, Stephanie D’Costa, Myrto Vlazaki, Olaoluwa Ayeko, Edward H. Arbe-Barnes, Casey B. Swerner

**Affiliations:** 1 St John’s College, University of Cambridge, St John’s Street, Cambridge, CB21TP UK; 2 St Peter’s College, New Inn Hall St, Oxford, OX1 2DL UK; 3 Gonville and Caius College, Trinity Street, Cambridge, CB2 1TA UK; 4 Newnham College, Cambridge, CB3 9DF UK; 5 Pembroke College, St. Aldates, Oxford, OX1 1DW UK; 6 Magdalen College, Oxford, OX1 4AU UK; 7 Institute of Cognitive Neuroscience, University College London (UCL), 17 Alexandra House, Queen Square, London, WC1N 3AZ UK

## Abstract

The 2015 Varsity Medical Ethics debate convened upon the motion: “This house believes nootropic drugs should be available under prescription”. This annual debate between students from the Universities of Oxford and Cambridge, now in its seventh year, provided the starting point for arguments on the subject. The present article brings together and extends many of the arguments put forward during the debate. We explore the current usage of nootropic drugs, their safety and whether it would be beneficial to individuals and society as a whole for them to be available under prescription. The Varsity Medical Debate was first held in 2008 with the aim of allowing students to engage in discussion about ethics and policy within healthcare. The event is held annually and it is hoped that this will allow future leaders to voice a perspective on the arguments behind topics that will feature heavily in future healthcare and science policy. This year the Oxford University Medical Society at the Oxford Union hosted the debate.

## Background

The types methods of cognitive enhancement are multifarious in their mechanism and plausibility [1]. However, compared to the abstract debate about enhancement in general, pharmacological manipulation via existing prescription drugs presents a very immediate ethical problem that requires attention.

The origin of the immediacy of this issue is twofold. Firstly, the problem concerns drugs which are already legally, safely and widely used amongst patient populations [2]: Ritalin/Adderall for Attention Deficit Hyperactivity Disorder (ADHD) and Modafinil for narcolepsy. Secondly, this method of enhancement is one that is actually in current use in an entirely unregulated fashion, by a significant proportion of the population. In the U.K. Ritalin and Adderall are considered class B drugs [3], punishable up to 5 years in prison for possession, and Modafinil is considered a prescription only medicine [4].

In the most recent large scale formal survey in The United Kingdom & Ireland [5], the lifetime use of prescription drugs that have purported cognitive enhancing functions without a prescription in students has been estimated at around 10 %. Alarmingly, a further 20.4 % have considered using such drugs, for which ‘lack of availability’ was sighted as the major reason for not using; suggesting the potential for a positive increase in the future. The most common of these, Adderall, Methylphenidate (Ritalin) and Modafinil are either obtained from friends or online. Users obtain these drugs most commonly with the aim of enhancing cognition (as opposed to offsetting sleep deprivation or enhancing mood). Whilst estimates of the prevalence vary, results from the United States (14 % of 381 respondents) [6] and Switzerland (7.6 % of 6275 students), demonstrate that non-prescription use of prescription only drugs for the purposes of cognitive enhancement poses a significant ethical problem.

It should be noted that this article limits its scope to the ethics of nootropic prescriptions on the NHS, rather than economic arguments about allocating resources to such drugs. We firstly discuss whether the use of these drugs can be considered medically appropriate for cognitive enhancement. Secondly, we explore the ethical implications of providing such drugs under prescription; analysing the tension between providing a safe mechanism for their use, and broader objections to ‘legitimising’ enhancement. We argue a prescription framework provides the best framework whilst also avoiding the ethical pitfalls that may arise from making such drugs freely available.

## Legitimate nootropic substances?

Before any claim can be made for the ethical benefits of allowing such drugs to be provided by prescription for nootropic purposes, we must satisfy the empirical question as to whether these drugs should be considered as nootropic. A meta-analysis [7] of 175 primary literature studies of Modafinil in humans demonstrated significant improvements in executive functions (most notably attention and memory) on both simple tasks and complex tasks. However, only 24 studies employed a double blind randomized control trial study design. Ritalin, in a meta analysis of 19 randomized control trials [8], was found to demonstrate a consistent effect specific to memory, but failed to demonstrate an effect for other executive functions. Repantis et al. also report an increase in attention for Modafinil amongst non-sleep deprived subjects in a meta-analysis of 31 RCT primary literature papers. Whilst neither meta-analysis demonstrated no consistent serious side effects, it should be noted that there is a potential for abuse of amphetamine like substances (such as Ritalin/ Adderall) [9]. In sum, there is a small but growing body of evidence to suggest that nootropic drugs can fulfil their potential as cognitive enhancing agents, and therefore, we argue that arguments that rest on dismissing their availability under prescription based efficacy are not sufficient.

## Right to access

These days, no one would doubt that we live in a very fast-paced society that is often highly pressurised. If this were to cause symptoms in a patient as a consequence, such as stress, a doctor or other professional would often advise the individual to try and make their life less stressful. However, we understand that the fast-paced nature of society is unlikely to change and therefore finding ways to cope is the best way forward. This leaves an opening for nootropic drugs to help cope with the demands of modern society.

Even if it is the case that certain nootropic substances may help with such demands, the question arises as to whether such substances should be available or whether legalisation should ban its use. The right of access to such drugs for a willing agent must be weighed against the effect of granting that right to society as a whole. Objections to the availability of cognitive enhancement usually centre on the creation of an uneven playing field, and the creation of a ‘burden to dope’ in the face of culture that promotes enhancement [10].

Do nootropic drugs create an uneven playing field? Whilst the use of cognitive enhancers clearly would provide some extra comparative benefit to a person, it does so in the background of a plethora of inequalities (economic, social, genetic, environmental) which can contribute to inequalities in cognitive function. Secondly, given that such substances are already being used (fairly widely) for their nootropic effects, the playing field is already tipped towards those who can afford them. By providing them under legal prescription, we aim to remove economic and legal barriers to their access.

The creation of a ‘burden to dope’ provides a more serious challenge to the availability of nootropic drugs, whether it be directly from another agent (such as an employer) or a more implicit burden to ‘keep up’ with other agents who are using cognitive enhancing drugs. However, to suggest that legal banning such substances would remove such a burden ignores the fact that this burden already exists within certain populations; in an environment of unregulated supply and lack of medical monitoring. Secondly, it naïvely assumes that by making such drugs available implies that they would be available unconditionally. If the provision of such drugs would be held to the same standards as other healthcare services, i.e. that of patient autonomy (free from external constraints and having relevant internal capacities to consent [11]), then it is unclear why cognitive enhancement should be considered distinct from other elective procedures such as plastic surgery. Therefore, we argue that, carefully monitored, the right to access of nootropic drugs should be advocated.

## Need for prescriptions

The demand for nootropic drugs and the current state of the law creates a situation whereby supply arises from a black market. A key benefit of nootropic drugs becoming available for prescription would be the licensing and regulation processes that they would therefore be subject to. For existing prescription drugs that are used without a prescription, such as Ritalin and Modafinil, this means that rather than purchasing from unregulated sources such as friends or the internet, drugs would become available from a legitimate source. This means someone taking a nootropic knows exactly what they’re taking, which is very difficult to qualify from an unregulated online source, and what the potential side effects could be. If a drug is prescribed from a physician the patient can be monitored to observe for adverse side effects as well as efficacy. A system where a doctor has ‘control’ over a patient’s usage of a cognitive enhancing drug is inherently safer than one where there is no experienced and professional input. The safety of individuals on the whole is of critical importance and by this means efficacy can also be monitored more efficiently on a widespread scale.

Creating a legal market for nootropics available under prescription would also attract investment and research into the field. Whilst we have highlighted the benefits of certain drugs on cognitive functions, the field is marked by a paucity of data on existing drugs in longitudinal studies. Particularly important here is the question of dosage guidelines for nootropic consumption: PET scans illustrated that 400 mg of modafinil had observable effects in parts of the brain involved in substance abuse and dependence [12], and therefore clearly appropriate guidelines need to be created for their consumption. However, such research will only be forthcoming upon legitimising such a use. Finally, it is worth noting that currently available nootropics have arisen from drugs intended for use for particular diseases. Opening up the field would allow research to begin on projects centred on pharmacological research of cognitive enhancement, rather using existing molecules for novel purposes (such as Ritalin or Modafinil).

## Conclusion

An environment currently exists in society where the use of nootropic drugs is widespread within certain groups of people. We argue the reality of the situation should be accepted and everything done to make it as safe and regulated as possible. By making these drugs available under prescription, we argue that we will remove the negative effects of a ‘black market’ of prescription drugs whilst also avoiding the potentials for abuse by making them freely available. Furthermore, we argue that by incorporating them into a formal medical framework, we will foster an environment which promotes research and hopefully produces better nootropics for the future.

## About the debate

After very persuasive arguments from both sides, the judges awarded victory to the Cambridge team (proposition) this year. A key area of contention was in the safety of the nootropic drugs, with Oxford arguing that regulation was undermined by the lack of solid data on the effects of such drugs taken for solely nootropic benefit. Cambridge argued, on the other hand, that the safety and efficacy of consumption would be improved by their availability under prescription from doctors, and that the state had a duty to provide assistance to those who legitimately required the drugs for nootropic related reasons.

## Abbreviations

(c): Captain
ADHD: Attention Deficit Hyperactivity Disorder
PET: Positron emission tomography
RCT: Randomized controlled trial
UCL: University College, London
